# Microinjection support system for small biological subjects

**DOI:** 10.1016/j.ohx.2020.e00103

**Published:** 2020-03-13

**Authors:** Yasuhiro Sugimoto, Keisuke Naniwa, Hitoshi Aonuma, Koichi Osuka

**Affiliations:** a Department of Mechanical Engineering, Osaka University, 2-1 Yamadaoka, Suita, Osaka 565-0871, Japan; b Research Center of Mathematics for Social Creativity, Research Institute for Electronic Science, Hokkaido University, Kita 12, Nishi 7, Kita-ku, Sapporo, Hokkaido 060-0812, Japan; c Japan Science and Technology Agency (CREST), Kawaguchi Center Building, 1-8 4-Chome, Honcho, Kawaguchi, Saitama 332-0012, Japan

**Keywords:** Injection system, Liquid solution injection, Sample preparation, Open source robotics, Image processing

## Abstract

In biological research, various experiments such as behavioral experiments and physiological ones are often conducted with pharmacologically treated animals. In such experiments, it is necessary to inject the same volume of solution into numerous small animals, such as insects to prepare several experimental subjects. However, repeating manual injections is burdensome, and it is also difficult to maintain injection quality and consistency. We have developed a microinjection system that can support and semiautomate the injections of small animals. The system consists of two cameras, a micromanipulator, a syringe pump, and a structural framework all operated from a personal computer to quickly inject the same volume of liquid solutions at the same position and depth into small animals. The microinjection system has sufficient extensibility for it to be used in a variety of applications.

## Graphical abstract

**Figure:**
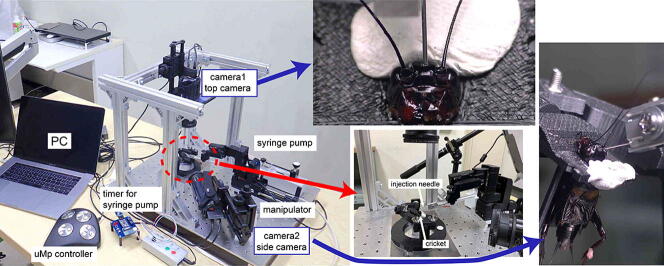

**Specifications table:**

**Table:** 

Hardware name	Microscale injection support system
Subject area	Biological Sciences
Hardware type	Biological sample handling and preparation
Open source license	Various
Cost of hardware	USD 3800 (except uMp manipulator system) + USD 12000(uMP manipulator system)
Source file repository	https://osf.io/3mtxk/

## 1 Hardware in context

Various biological research experiments, such as behavioral and physiological experiments, are typically conducted with pharmacologically treated animals. Such experiments are also being conducted on insects [1]. In the current study, inspired by the unique hunting behavior of *Ampulex compressa* [2], [3], a microinjection experiment was conducted to prepare a “zombie cricket,” which has a minimal central nervous system to produce a variety of gaits by pharmacologically blocking of the cricket’s nervous system. Because it is expected that the adaptive locomotion mechanisms of walking animals are revealed by investigating various behaviors of zombie crickets [2], it is important to be able to prepare several such crickets.

Although it is first essential to develop an effective method of zombifying crickets, it is also crucial to prepare numerous zombified crickets whose degree of zombification is uniform to conduct gait measurement experiments on many such crickets. Therefore, it is necessary to insert an injection needle at the same position and depth and inject the same volume of zombifying solution into each cricket, a complicated operation requiring demonstrable repeatability. Currently, we manually inject crickets by following a conventional method similar to those used by other biological researchers, which inevitably leads to some degree of variation in zombification and subsequent research results. Moreover, it is difficult to manually inject a solution into a specific part of the cricket’s brain, because it is quite small. In addition, manually injecting many small biological subjects, such as crickets, increases the risk of sticking the needle into one’s own hands. Therefore, it is more pragmatic to develop a microinjection system that supports and semiautomates safe injection into small animals under many experimental conditions. Because it is quite common to inject liquid solutions into small animals such as insects, such a system is useful not only for our project but also for other biological experiments; thus, the development of such a system should have a significant ripple effect. Although several microinjection systems have already been developed, most have been for injection into cells [4], [5], [6] or very small animals such as *C. elegans* [7]. There are also some patents [8], [9] and commercial products [10] available for injection systems. However, to the best of our knowledge, an integrated injection system combining image processing and syringe-pump operation has not been developed yet. Furthermore, most of the previously proposed systems utilize a microscope. Although a high-magnification clear image can be obtained from a microscope, the distance between the lens and the target was small. In order to inject into an insect-sized target, it is necessary to grasp the 3D position and posture of the target. Although stereoscopic vision is possible with a stereo microscope, the angle of field in the height direction is insufficient for such applications. Therefore, there are various restrictions on the posture of the target or manipulator movements.

Therefore, we have designed and developed a microinjection support system for small biological subjects such as insects. The design requirement of the system are as follows; (1) The system must be able to inject a certain volume of solution at the same position and depth into small creatures such as insects. (2) The system must be able to efficiently inject biological subjects in almost the same length of time as manual injections; that is, several minutes per injection target. (3) The system must show sufficient extensibility for it to be used not only in our project but also in other micromanipulations including microinjections.

## 2 Hardware description

From the system design requirements, the following design specifications were obtained:

• Measure the three-dimensional (3D) position of the needle tip using multiple cameras
• Inject a certain quantity of solution using syringe pump and timer
• Ensure sufficient working distance to facilitate mounting/dismounting of injection subjects and to reduce the restriction of the target posture and manipulator movement
• Achieve semiautomatic injection operation by controlling the entire system with a personal computer (PC)
• Show sufficient extensibility

**Fig. 1:**
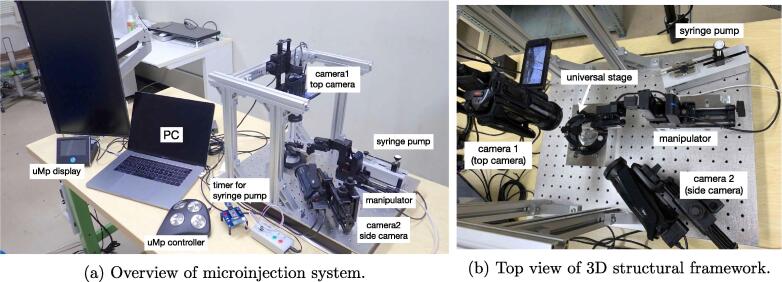
Assembled microinjection support system.

Based on the design specifications, the microinjection support system was developed for small biological subjects. Figs. 1a and 1b show the assembled system, which consists of the following:

• Two video cameras and corresponding PC video/still-image-capture devices
• A micromanipulator system for accurate needle operation
• A syringe-pump system for consistent injection of certain solution quantities
• A framework for fixing video cameras and the manipulator
• A universal stage for mounting/holding injection subjects
• A PC for controlling the entire system

The details of each system component are described below.

### 2.1 3D structural framework and universal stage

For the proposed microinjection support system to be effective, the operation time for injections must be several minutes per biological subject. To meet this requirement, it is necessary to reduce the time required for preparing each subject for injection. In particular, it is essential to minimize the time required to focus video cameras, adjust injection-target postures, and mount/dismount injection targets. As regards focusing video cameras, autofocus did not work well because macrolenses were regularly used for microradiography experiments. Instead, video-camera positions were finely adjusted by attaching mag sliders (A7 and A9) to the video cameras. Next, injection-subject postures must be adjusted because it is difficult to finely adjust the angles and directions of needles inserted into biological subjects by the manipulator. To facilitate adjustment, a universal stage showing three rotational degrees of freedom (i.e., pan, tilt, and roll) was designed to mount the injection targets instead of using a commercially available automatic stage. The target size is expected to be several tens of millimeters to several millimeters. The universal stage itself can be easily removed from the 3D structural framework, and it can also remove the catcher, which mounts injection subjects. Therefore, the injection subjects can be mounted/dismounted outside the 3D structural framework because mounting them inside it is difficult. Mag sliders and the manipulator were fixed with self-made adapters to the surface plate (A1) and the 3D structural framework, which was assembled on the plate. The universal stage was placed on the substage, which was fixed to the surface plate. Adapters and the substage were prepared by cutting aluminum (A5052) blocks and fixing them tightly to suppress vibration, thereby not affecting video/still-image capture or manipulator operation. Using the 3D structural frame, the universal stage, and a camera equipped with a close-up lens as described in Section 2.2, the working distance can be enlarged. As a result, not only the work efficiency is improved, but the target posture and manipulator movement constraints can also be reduced. This feature is significant not only in the realization of microinjection but also in the extension to other micromanipulations.

### 2.2 Video/still-image capture

Two video cameras (JVC GV-R470) were fixed to the top (i.e., camera 1) and side (i.e., camera 2) of the universal stage to measure the 3D needle-tip positions relative to the injection subjects. When the injection target is expected to be several tens of millimeters to several millimeters, like cricket, it is necessary to observe directly using a horizontal camera to confirm the target’s posture and the needle insertion angle. In a stereoscopic vision with a stereo microscope, the angle of view in the height direction is insufficient. Furthermore, a system using two microscopes is expensive. Therefore, the proposed system consists of two cameras instead of a stereoscopic microscope. Close-up lenses (Raynox Corp., DCR-250) were attached to video-camera lenses because the macrophotographic functions of the video cameras were insufficient. Using close-up lenses and camera zoom functions, it is possible to ensure sufficient distance between cameras and biological injection subjects. Therefore, this configuration enables high magnification while ensuring 100 to 150 mm of working distance. Videos/still images output from the HDMI output terminal of each camera were captured on the PC by a video/still-image-capture device (Blackmagic Design UltraStudio Mini Recorder).

### 2.3 Manipulator

A micromanipulator (Sensapex uMp-4, C12) was used to precisely control the injection-needle position. The manipulator shows a quad axis; that is, it shows four degrees of freedom (i.e., needle direction (w-axis) in addition to x-, y-, and z-axis directions). By ultrasonic driving, the resolution and repeatability of position control of the manipulator are 5 nm and 100 nm respectively [11]. Therefore, it is easy to control the position and depth of needle insertion. The needle was attached to the manipulator by a needle holder. The manipulator system consists of a uMp manipulator, controller, and display and can be operated not only manually from the uMp controller but also remotely from a PC using the software development kit (SDK) released by the manufacturer. Moreover, the manipulator can be operated using both together. In the software developed to control the whole system, manual and PC operation can be switched quickly according to the situation and operation mode to achieve semiautomatic injection operation.

### 2.4 Syringe pump

A syringe pump (AS ONE MR-1, D2) and a controller (AS ONE CT-10, D1) were used to consistently inject a certain volume of liquid solution into biological subjects. As the controller can set syringe-pushing speed, it is possible to determine, based on syringe diameter, the operation time required for injecting a certain volume of solution into a subject. Because the controller itself was not equipped with a timer or directly interfaced with the PC, a self-made timer circuit produced using Arduino (an open-source electronics platform) was added to the controller to turn the syringe pump on and off. In addition, timer on/off modes and set times can be remotely controlled from a PC by serial communication.

### 2.5 Control software

The control software used to integrate each device was developed in C++ on a PC (15-inch MacBook Pro). From video/still-image-capture devices, videos/still images were captured to the PC using Blackmagic DeckLink SDK released by the manufacturer [12]. Because the SDK alone cannot capture videos/still images directly using OpenCV, an open-source computer vision library, the developed software also used a wrapper library for this task. Using threading building blocks (TBB) pipeline processing (Intel®Corp), two full-high-definition (HD, 1920 × 1080) still images can be captured at 30 fps. This is the upper frame-rate limit for capturing still images on video/still-image-capture devices. Captured images can also be displayed on the PC with almost no delay. As a result, system operation was not hindered while viewing captured videos/still images. The injection timer control on Arduino and the position control of the manipulator by the uMp controller are executed independently of the control on PC. Besides, parallel processing was also implemented such that the control loop for displaying captured images and the control loop for handling keys and mouse inputs and sending commands to Arduino and the uMp controller are executed in parallel. Therefore, the operation of the manipulator and syringe-pump operation was not disturbed by the capturing and displaying of still images.

### 2.6 Microinjector extensibility

Although the proposed microinjection support system was originally developed to facilitate cricket-head/brain injections in our project, its application can be further extended. By changing the removable catcher of the universal stage, various biological subjects can be mounted, and subject postures can easily be adjusted because of the universal-stage configuration. Therefore, multiple biological subjects can be injected from various target positions. Furthermore, because still images were captured from the video cameras using OpenCV, multiple-image processing systems can be implemented using many software libraries included in OpenCV, thereby achieving automatic operation by combining image processing with manipulator and syringe-pump operation. In addition, the system can be used not only to inject solutions into biological subjects, but also to conduct electrophysiology experiments for measuring the myogenic potential or neuron activity of biological subjects by attaching electrodes to manipulators. Therefore, the proposed microinjection support system shows sufficient extensibility to other micromanipulations in addition to injections.

## 3 Design-software files

Table 1 lists the provided design-software files required to fabricate and operate the proposed microinjection support system. The following is a brief summary of the contents of the files:

universal_stage.zip Computer-aided design (CAD) files required for universal stage, including Autodesk Inventor part file and stereolithography (STL) file required for 3D printing.
machining_drawing.zip CAD files required for camera and manipulator adapters, including Autodesk Inventor part file and mechanical drawing.
msis2_cad.zip CAD files indicating the positional relationship of 3D structural framework, cameras, and manipulator, including Autodesk Inventor part file.
needle_holder_cad.zip CAD files required for needle holder operation, including Autodesk Inventor part file and mechanical drawing.
injectionTimer_pcb_design.zip Eagle design files required for injection timer printed circuit board (PCB).
msis2.zip C++ source codes for controlling the whole system.
blackmagic-opencv-wrapper.zip C++ source codes required for controlling whole microinjection support system.
injectionTimer_arduino.zip Arduino firmware required for controlling syringe pump
BOM_injectionTimer.xlsx Bill of materials (BOM) Excel spreadsheet required for injection timer PCB.

**Table 1:** Design-software files and associated relevant information.

Design filename	File type	Open source license	File Location
univeral_stage_stl.zip	STL files	CC BY-NC 4.0	https://osf.io/mk3qt/
machining_drawing.zip	CAD files	CC BY-NC 4.0	https://osf.io/xs26v/
msis2_cad.zip	CAD files	CC BY-NC 4.0	https://osf.io/mrfkc/
needle_holder_cad.zip	CAD files	CC BY-NC 4.0	https://osf.io/ysfj3/
injectionTimer_pcb_design.zip	PCB files	MIT License	https://osf.io/fqpe7/
msis2.zip	C++ code	MIT License	https://osf.io/j8c4k/
blackmagic-opencv-wrapper.zip	C++ code	Apache 2.0 License	https://osf.io/9pjxq/
injectionTimer_arduino.zip	Arduino firmware	MIT License	https://osf.io/xzgq9/
BOM_injectionTimer.xlsx	Excel spreadsheet	CC BY-NC 4.0	https://osf.io/6wjuk/

## 4 Bill of materials

Table 2 lists the provided bill of materials required to build proposed microinjection support system.

• A5, A6, A8, and A10 must be machined according to the CAD drawings included in Machining_drawing.zip.
• Although Velbon Super Mag Slider(A7) has two stages, only one of the two stages (in z-direction) was used.
• B1, B2, B3, B5, and B6 were prepared by a 3D printer according to STL files included in universal stage.zip
• D4 and D5 must be machined according to the CAD drawings included in needle_holder_cad.zip.
• ^†^ indicates the component can be replaced with equivalent device.

**Table 2:** Bill of materials required to build the proposed microinjection support system.

Designation	Component	Number required	Unit Cost (USD)	Total cost (USD)	Material Source	Material type
*3D structural framework*						
A1	Thin Steel Honeycomb Optical Surface Plate C0405T	1	627	627	AS ONE	
A2	Aluminum Frame (6 Series 320 mm)	7	2.09	14.63	MISUMI	Aluminum
A3	Hard Bracket L	12	2.25	27	MISUMI	Aluminum
A4	Hard Bracket LG	4	5.04	20.16	MISUMI	Aluminum
A5	Aluminum free plate (PNLNP-150-100-30) for Substage	1	21.27	21.27	MISUMI	Aluminum
A6	Aluminum free plate (PNLNP-100-40-15) for Camera 1 adapter	1	11.55	11.55	MISUMI	Aluminum
A7	Velbon Super Mag Slider	1	78	78	Amazon	Magnesium
A8	Aluminum L-angle plate (LASA7550-50-10) for Camera 2 adapter	1	19	19	MISUMI	Aluminum
A9	Velbon Super Mag Slider	1	78	78	Amazon	Magnesium
A10	Aluminum free plate (PNLNP-150-70-20) for Manipulator adapter	1	22.45	22.45	MISUMI	Aluminum
A11	Hex Bolt, 3/8, 3/4 in.	2	2.18	4.36	MISUMI	Stainless steel
A12	Hex Bolt, 1/4, 3/4 in.	2	1.49	2.98	MISUMI	Stainless steel
A13	Hex Bolt, M6, 20 mm	11	0.11	1.21	MISUMI	Stainless steel

*Universal stage*						
B1	Stage base	1				ABS
B2	Stage arm	2				ABS
B3	Stage arm rod	1				ABS
B4	Small Deep Groove Ball Bearings - Double Shielded with Flanged	2	4.45	8.9	MISUMI	Stainless steel
B5	Gripper	1				ABS
B6	Catcher	1				ABS

*Electrical equipment*						
C1	JVC, Everio R GZ-R470-H	2	289	578	Amazon	
C2	Raynox, Super MacroScan Conversion Lens DCR-250	2	68	136	Amazon	
C3	Stepup Ring^†^ 37 mm → 43 mm	2	4	8	Amazon	
C4	Kenko, PRO1D WIDE BAND Circular PL filter^†^ 49 mm	2	32	64	Amazon	
C5	Blackmagic Design, UltraStudio MiniRecorder	2	167	334	Amazon	
C6	Thunderbolt 3(USB-C) – Thunderbolt 2 Universal Serial Bus Adapter^†^	2	63	126	Amazon	
C7	Thunderbolt Cable^†^ (0.5 m)	2	38	76	Amazon	
C8	Anker, PowerPort^†^ 5(USB-C) Power Deliver	1	36	36	Amazon	
C9	Micro USB cable^†^ (2 m)	3	5	15	Amazon	
C10	Satechi USB-C Hub^†^	1	77	77	Amazon	
C11	Thunderbolt 3(USB-C): Ethernet Adapter^†^	1	18	18	Amazon	
C12	Sensapex uMp micromanipulation system	1	12000	12000	Intermedical Co.,Ltd	

*Syringe pump and needle holder*						
D1	Syringe Pump Remote Controller CT-10	1	570	570	AS ONE	
D2	Syringe Pump Remote Controller Drive Part MR-1	1	725.45	725.45	AS ONE	
D3	Hamilton, Gastight Syringe 1705LT	1	87.27	87.27	AS ONE	
D4	Aluminum round rod(537) for Needle holder 1	1	2.44	2.44	MISUMI	Aluminum
D5	Aluminum free plate(H-PNLNN-70-10-4) for Needle holder 2	1	5.27	5.27	MISUMI	Aluminum

## 5 Build instructions

### 5.1 3D-structural-framework assembly

**Fig. 2:**
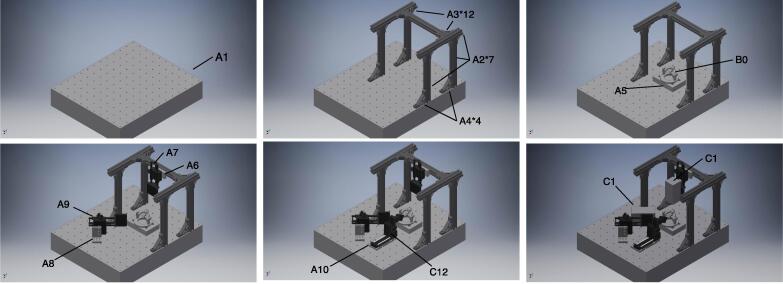
Schematic representation of the proposed microinjection system assembly. 1. Surface-plate (A1) preparation. 2. Assembly of aluminum frames (A2) with hard bracket L (A3) and fixture on surface plate with hard bracket LG (A4). 3. Attachment of substage (A5) with M6 20-mm hex bolts (A13) and installment on universal stage on substage. 4. Attachment of camera adapter 1 (A6) and camera adapter 2 (A8) to super mag sliders (A7 and A9) with 3/8”(A11) and 1/4”(A12) hex bolts and fixture of adapters to frame and surface plate with M6 20-mm hex bolts (A13), respectively. 5. Attachment of manipulator(C12) with manipulator adapter(A10). 6. Attachment of cameras to Super Mag Sliders.

Fig. 2 shows a schematic of the microinjection-system assembly. The hardware of the proposed system consists of an aluminum structural framework assembled on a surface plate (A1) and equipped with the universal stage, two video cameras (C1), and the manipulator (C12) by adapters and a substage. The 3D structural framework was assembled from aluminum frames (A2) and brackets (A3 and A4). Before the 3D structural framework can be assembled, adapters and the substage must be prepared by machining aluminum-free plates (A5, A6, A8, and A10). Furthermore, it is better to equip the cameras with conversion lenses (C2) and linear polarizing (PL) filters (C4) via stepup rings (C3) before attaching the cameras to the super mag sliders. Fig. 4a shows the completely assembled 3D structural framework.

### 5.2 Universal-stage assembly

**Fig. 3:**
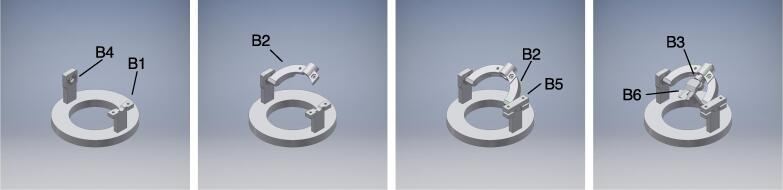
Schematic representation of universal stage assembly. 1. Fit bearing (B4) to stage base (B1). 2. Plug stage arm (B2) into bearing. 3. Mount the stage arm (B2) and tighten it with screws and nuts by gripper (B5). 4. Plug stage arm-rod (B3) with catcher (B6) and tighten with screws and nuts.

**Fig. 4:**
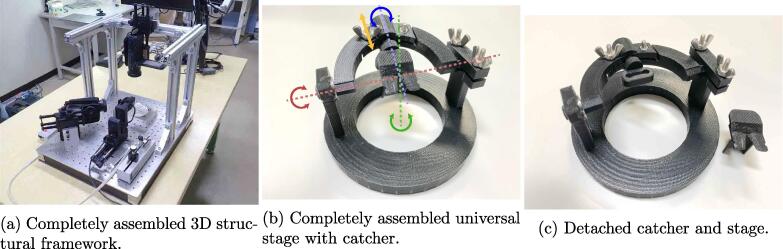
(a) Completely assembled 3D structural framework. (b) Completely assembled universal stage. (c) Catcher can be detached from the stage. Because the stage shows various degrees of freedom in pan (green), tilt (red), roll (blue), and distance (yellow), catcher posture and position can be easily adjusted. Moreover, because three axes (i.e., pan, tilt, and roll) intersect at one point, changing any of these angles does not affect intersection position. The intersection was designed to match the position of the target on catcher. Therefore, once the focus is adjusted to the target, the focus does not need to be readjusted even if pan, tilt, or roll angles change. This structure will facilitate injections.

Fig. 3 shows a schematic of the universal-stage assembly. The universal stage was fabricated using 3D-printed parts, ball bearings, screws, and nuts. The screws were not overly tightened because it is better to let the arm parts rotate slightly for fine adjustment of target position and posture. Fig. 4b shows the completely assembled universal stage.

### 5.3 Electrical equipment assembly

**Fig. 5:**
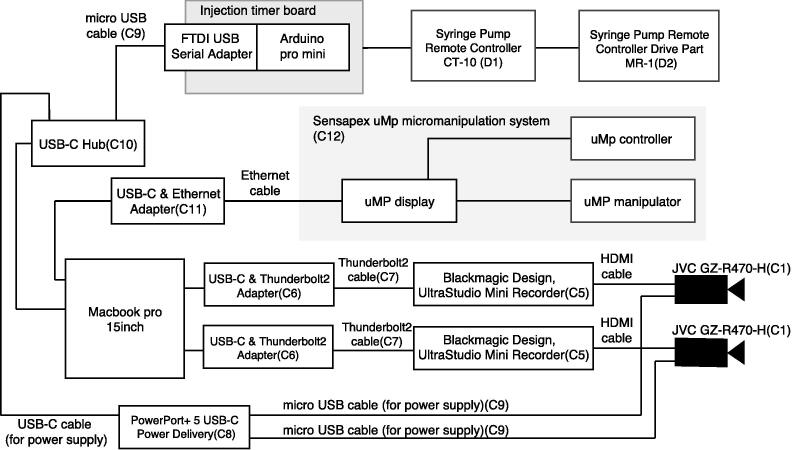
Electrical equipment configuration. CT-10 (D1) and MR-1 (D2) are connected with cables attached to CT-10 (D1). CT-10 (D1) and the injection-timer control board are connected with self-made cables (See Section 5.4). Ethernet and HDMI cables are attached to the manipulator system (C12) and video cameras (C1), respectively.

Fig. 5 shows configuration of the electrical equipment. The electrical equipment of the entire system consists of the image-capture subsystem (i.e., video cameras and video/still-image-capture devices), manipulator system, syringe-pump subsystem (i.e., pump, controller, and timer), and control PC. The figure shows all the devices and connections between them and power-supply lines.

### 5.4 Syringe-pump and injection-timer assemblies

**Fig. 6:**
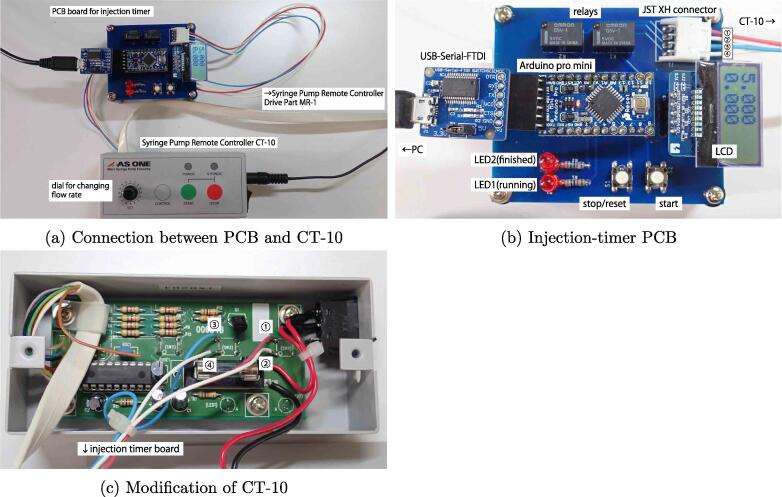
Injection-timer system developed with syringe-pump remote controller CT-10. (a) Arduino controls any relays to switch CT-10 on/ff based on timer state. (b) LCD displays timer set and elapsed times. Two tactile switches can start/stop timer. Two LEDs show running/finished timer states. (c) PCB and CT-10 are connected by four wires. Wires 1 and 2 and wires 3 and 4 are soldered at SW3 and SW4 on CT-10 PCB, respectively.

Fig. 6a shows the syringe-pump and injection-timer assemblies. Fig. 6b shows custom PCB of the injection timer, identifying the main components and interfaces. The eagle design files are included in injectionTimer_pcb_design.zip. Although the PCB shown in the figure was fabricated by a PCB manufacturer [14] and parts of the PCB were manually soldered, it may be implemented on a prototype PCB universal board or breadboard because it has only few components. The syringe-pump remote controller CT-10 does not have an external interface, so it must be slightly modified by soldering four wires from the PCB to the CT-10 control board, as shown in Fig. 6c to externally control the on/off button. The injection timer uses a 5 V, 16 MHz Arduino pro mini 328, and the PC and Arduino pro mini 328 were connected by a FTDI USB serial Adapter. It is required to flash the injectionTimer.ino file included in the injectionTimer_arduino.zip with an Arduino IDE. CT-10 has a dial that can change the injection flow rate by changing the syringe-pushing speed. For specific flow rates, refer to the instruction manual attached to CT-10.

**Fig. 7:**
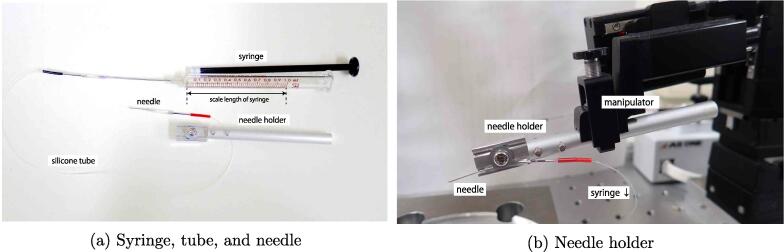
(a) Syringe and injection-needle setup. The silicone tube (D6) is attached to syringe (D3), and the needle is inserted into the other side of the tube. (b) The needle holder is assembled from machined needle holders 1 (D4) and 2 (D5) with screws. The needle connected to the silicone tube is attached to the needle holder with screw and washer.

Fig. 7a shows the connections among the syringe (D3), silicone tube (D6), needle, needle holders (D4 and D5), and manipulator. The needle from which the needle hub was removed was inserted into the tip of the tube. The syringe and needle were connected by a silicone tube, and the needle was then attached to a needle holder, which was subsequently attached to the manipulator(Fig. 7b). The syringe was mounted on the syringe-pump remote controller drive MR-1 (D2).

### 5.5 Software setup

msis2.zip is an archived source of software code that controls the entire microinjection system, including the camera image-capture devices, manipulator, and injection timer. The software depends on the following libraries, which must be installed on the PC before building the software.

1. Blackmagic Desktop Video software
2. Blackmagic Desktop Video SDK
3. Blackmagic-opencv-wrapper
4. Sensapex uMp SDK
5. OpenCV
6. Boost C++
7. AntTweakBar
8. TBB
9. GLFW
10. CMake

With the exception of the Blackmagic Desktop Video software (1), Blackmagic Desktop Video SDK (2), blackmagic-opencv-wrapper (3), and Sensapex uMp SDK (4), the software should be installed by homebrew on MacOS.

$ brew install opencv boost anttweakbar tbb glfw cmake

Blackmagic Desktop Video software distribution [12] provides system libraries for MacOS to capture images from Blackmagic Design UltraStudio Mini Recorder. After the Desktop Video software has been downloaded and installed on a PC, Blackmagic Media Express application, which is included with the Desktop Video software, should be checked to determine whether the device works without any problems.

Sensapex uMp SDK for uMp manipulator system can be downloaded from the Sensapex website [13]. After extracting the downloaded archive, it can be installed using the following commands:

$ cd extracted_dir/umpsdk/src
$ make -f Makefile.osx
$ sudo make install
$ cp ../libump.h/usr/local/include

Do not forget to copy libump.h to system include dir(/usr/local/include) because Makefile included in the SDK only copies built libraries.

Next, download and extract msis2.zip to a working directory, and download blackmagic-opencv-wrapper, extract it in the msis2 directory, and rename the extracted directory “DeckLink.” Finally, download Blackmagic Desktop Video SDK from the Blackmagic website [12] and extract it to the DeckLink directory. Create a symbolic link named **bmdsdk** to the extracted directory. The structure of the working directory thus becomes as follows:

**Figure:**
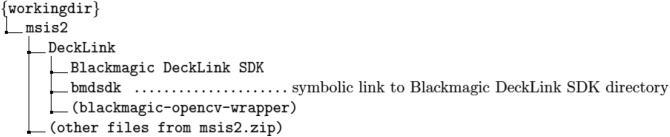

After dependent libraries have been prepared, the software for the entire system can be built with cmake. If the build process finishes without any problems, an executable file named “msis2” will be generated.

$ cd workingdir/msis2
$ cmake.
$ make

## 6 Operating instructions

The proposed microinjection system is externally controlled from the PC using the software built in Section 5.5. After turning on the camera, manipulator, and syringe pump, start the software on the command line as follows:

$ cd workinagdir
$./msis2

**Fig. 8:**
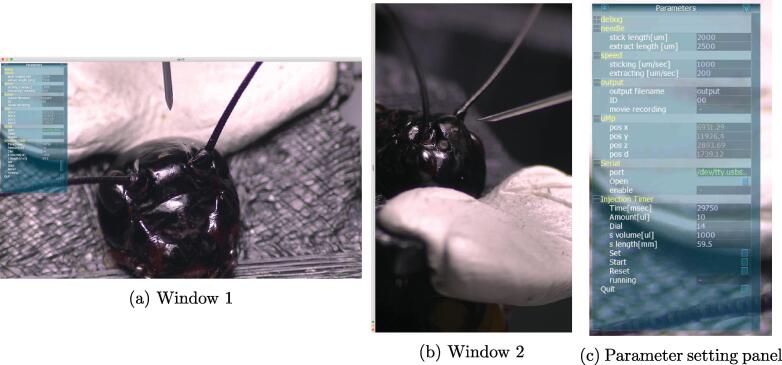
Screenshots of active injection program. (a) and (b) Windows 1 and 2 show real-time images captured from video cameras 1 and 2, respectively. (c) On parameter-setting panel, various parameters can be changed. The state of uMp manipulator is also shown.

Fig. 8 shows screenshots of the active program. Control software creates windows 1 and 2 to show real-time images captured from cameras 1 and 2, respectively. Window 1 also contains a graphical user interface (GUI) panel for setting injection, manipulator, and output-recording parameters (Fig. 8c). Before any injections are started, some settings must first be adjusted using the panel.

• Set the lengths of needle stick and extraction. The default stick and extraction lengths were 2000 and 2500 μm, respectively.
• Set the speeds of needle stick and extraction. The default stick and extraction speeds were 1000 and 200 μm/s, respectively.
• Select a serial port connected to the injection timer, and open the port. The name of the serial port usually becomes “/dev/tty.usb***” on MacOS.
• Set the syringe parameters.
  1. Input the syringe volume and corresponding scale length (See Fig. 7a).
  2. Enter the dial number set in the syringe-pump controller CT-10 (See Fig. 6a).
  3. Enter the injection volume.
  The injection times are automatically calculated from these parameters. The default settings are based on injecting 10 μl for about 30s using a Hamilton Gastight Syringe 1705LT (D3).
• (optional) If it is necessary to record movies during an injection, set a file name and ID. The filenames of recorded movies are determined by output filename, ID, date, and time. It is recommended that the ID of experiment subject is used as the ID for the movie file. If the movie-recording button is enabled, the movie will start recording automatically. Movie files are saved in the workinagdir/movies subfolder.

After the foregoing parameters have been set, injections can be started. The standard injection procedure is as follows.

1. Mount a biological subject, such as a cricket, on the universal stage.
2. Adjust the position and posture of the subject and then adjust the camera focus.
3. Use the uMp controller to move the needle tip to the point where the needle will pierce, based on images displayed on the control PC.
4. Press the “s” key to stick the needle.
5. Press the “start” button on the GUI panel to start the injection.
6. When the injection is finished, press the “e” key to extract the needle.
7. Move the needle away from the subject.
8. Dismount the subject.

Currently, the proposed system only uses OpenCV for displaying captured images. One of the reasons is that a manual operation may be faster than the automated operation because the injection target is visible. By using image processing libraries included in OpenCV, some of the injection procedures may be automated. And, by switching between automatic operation and manual operation properly, the total required time will be able to be reduced. Implementing image processing using OpenCV for reducing the operation time and injection into hard-to-see targets is an issue for the future.

## 7 System validation and characterization

We conducted four verification experiments to confirm the effectiveness of the constructed microinjection system. In the first experiment, the position and depth of the injection needle were verified. In the second experiment, the volume of liquid solution injected was confirmed. In the third experiment, liquid solution was injected into a cricket head as an example of injection into an actual biological subject. Finally, we verified whether the proposed system could make a zombified cricket.

### 7.1 Position and depth of injection needle

**Fig. 9:**
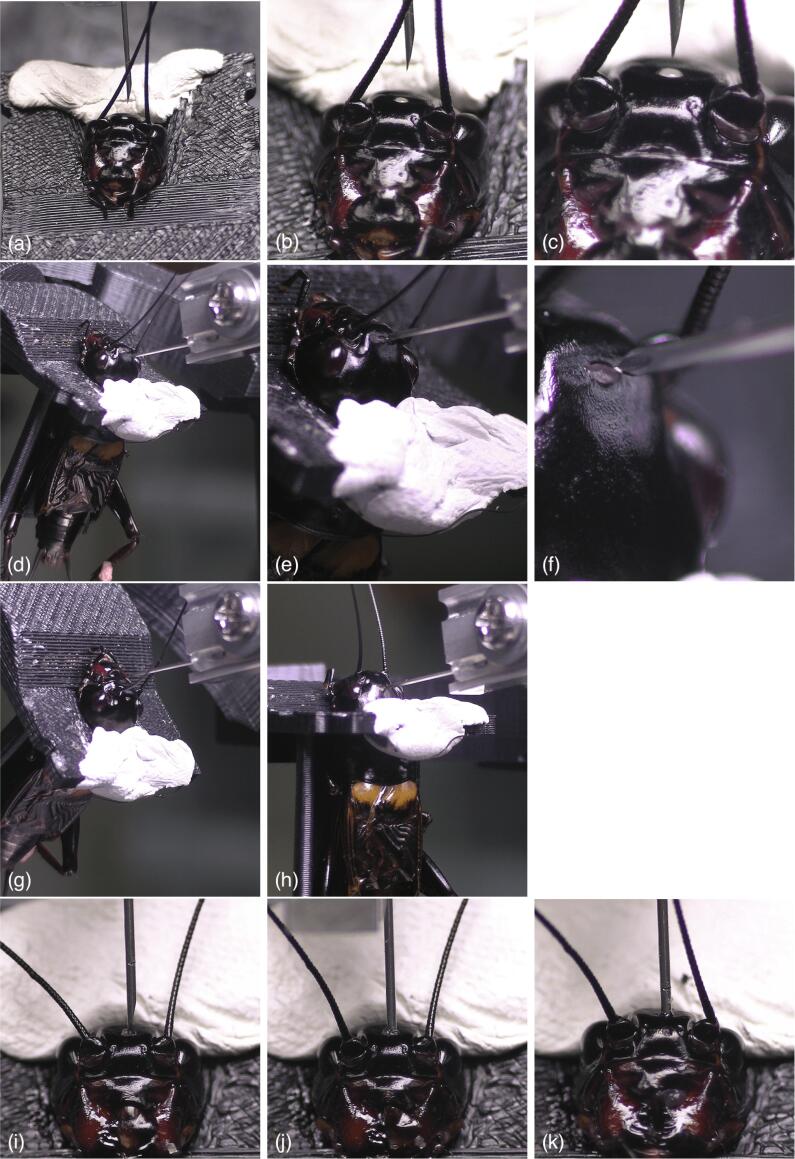
Validation experiment 1. (a, b, c) Captured images from camera 1 while changing photographing magnifications. (d, e, f) Captured images from camera 2 while changing photographing magnifications. (g, h) Captured images while changing the posture of the target. (i) Marked injection needle. (j, k) Captured images when the marked injection needle were stuck 3000 μm and 2000 μm respectively.

In verification experiment 1, it was confirmed that the injection needle could be stuck at the specified depth in the target direction at the target position. To stick the needle at the target position in the target direction, it is first necessary to grasp the position and posture of the injection target relative to the needle in real-time. In the constructed microinjection system, these are achieved by the images captured from two video cameras. Figs. 9(a)~(f) show images captured from video cameras while changing the photographing magnification. These figures show that the needle tip and the posture of the needle from the injection target can be confirmed at various photographing magnifications. The position of the needle tip can be controlled from the uMp manipulator controller. Further, the relative posture of the needle to the injection target can also be adjusted by changing the tilt angle of the universal stage. Figs. 9(g) and (h) show an example of changing the posture of the injection target. Therefore, in the constructed microinjection system, the position and posture of the injection target relative to the injection needle can be grasped in real time by adjusting the photographing magnification and focus of the camera according to the injection target. Subsequently, it is possible to stick the needle in the target direction at the target position based on the needle tip position and posture.

The sticking of the needle at the specified depth is realized by the position control of the uMp manipulator controller system. As described in Section 2.3, the manipulator system is capable of highly accurate position control for each axis. It is possible to stick the needle at the target depth by sending commands to the uMp manipulator controller from the PC indicating how much the manipulator is driven in the w-axis, which is the direction of the needle. We also conducted another experiment to verify the actual sticking depth. In this experiment, the needle that was marked as a reference for position measurement was stuck at the injection target(Fig. 9(i)). The distance from the mark to the needle tip was 3060 μm. Fig. 9(j) shows the result when the length of the needle stick on control software was set as 3000 μm, and the marked needle was stuck. As shown in the figure, the needle penetrated to the position of the mark on the needle. The needle was stuck at an accuracy of ±0.1 mm or less for the specified distance. Fig. 9(k) shows the result when the length of needle stick on the control software was set as 2000 μm. From the relationship between the actual length from the needle tip position to the mark and the distance on the image, the depth of the sticking was calculated to be 2046 μm. From these results, it is verified that the injection needle can be stuck to the target with a precision of ±0.1 mm or less for the specified distance.

Here, the accuracy of the needle sticking depth confirmed in the verification experiment is merely ±0.1 mm, and it is considered that more accurate needle position control is possible based on the manipulator specifications [11]. Moreover, the camera can zoom up to 40 times, but about 10 times zoom was only used in the verification experiment. There are higher magnification close-up lens. Therefore, higher resolution images can be acquired. From these specifications of the manipulator and cameras, the proposed system can handle targets smaller than crickets by changing the universal stage catcher (B6) and using a thinner needle. On the other hand, the mechanical parts (the universal stage and needle holder) of the system may not have sufficient rigidity to handle targets with a body length of 1 mm or less. The low rigidity causes insufficient holding of the target or the vibration of targets. The smaller the target, the higher these effects. In that case, it is necessary to redesign the mechanical parts.

### 7.2 Volume of liquid solution injected

**Fig. 10:**
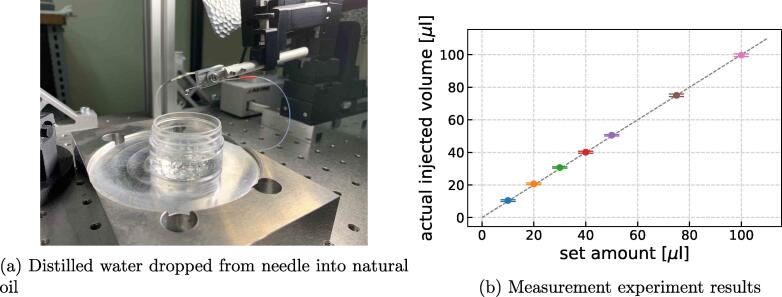
Validation experiment 2. (a) Distilled water was dropped from the injection needle into natural oil by the syringe pump and timer, and weights of water droplets were measured five times for each injection setting while increasing the setting from 10 μl to 100 μ. (b) Graph showing mean ± standard deviation of droplet weights measured for each setting.

Next, we verified whether the volume of solution set by the control software could be actually injected. In the experiment, the syringe-pump timer was externally operated from the control software, and distilled water was dripped into natural oil (Fig. 10a). The increase in oil mass was measured after water was injected, and the volume of water injected from the needle was calculated accordingly using the specific gravity of water at room temperature (25 °C). Fig. 10b shows experimental results. In this experiment, injections were repeated five times at each setting while changing the injection volume. In each injection, the same volume of distilled water as set on the control software had actually been injected, and any discrepancy between the set and actually injected volumes was negligible. From the result, it can also be verified that 10 μl injection is possible. From the specifications of the timer implemented by Arduino and the syringe pump controllers MR-1, it is considered that a smaller amount of injection may be possible. Verification of the smallest amount of injection possible will be a future issue.

### 7.3 Injection into biological subject

**Fig. 11:**
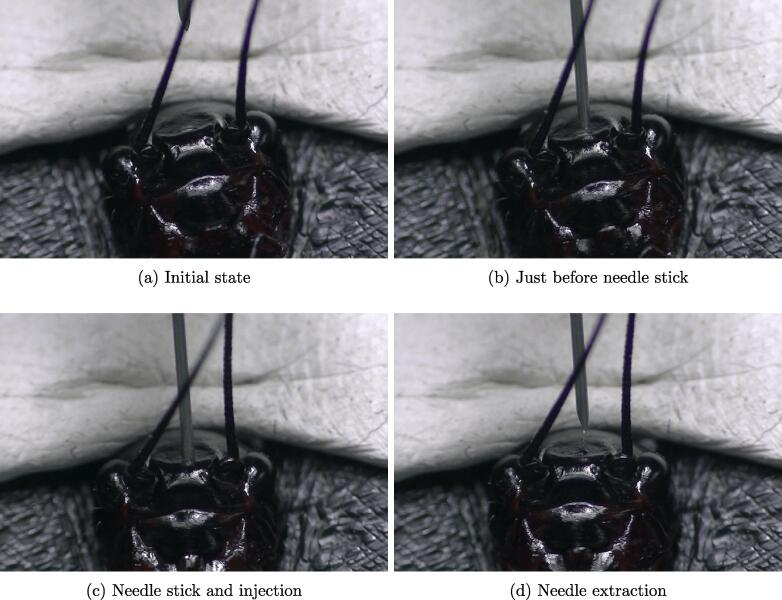
Images captured during verification experiment 3: (a) cricket mounted on universal stage, (b) needle tip moved using uMp controller to point where needle will stick, (c) needle stick and injection of 10 μl of liquid solution into cricket head, and (d) needle extraction.

Injection of the solution liquid into an actual biological subject was verified by injecting distilled water into a cricket head. Fig. 11 shows each injection step according to the injection procedure described in Section 6. As described in Section 6, some of the procedures can be automated by using image processing with OpenCV. However, the operation of moving the tip of the needle to the point where the needle will pierce was manually performed because the stemma of the cricket head, which is the target of the needle tip, is quite clearly visible. In this experiment, 10 μl of distilled water was injected into the cricket head. The figure clearly shows that the needle smoothly pierced the cuticle on the cricket head and that none of the injected water leaked out when the needle was extracted. In addition, we repeated the injection five times using 10 μl of cricket physiological saline on different cricket heads, and almost none of the injected cricket physiological saline leaked from any of the cricket heads when the needle was extracted. These results show that the constructed microinjection system was sufficiently effective as an injection support system.

### 7.4 Zombification of cricket

Finally, another verification experiment was conducted to verify whether the proposed system could prepare a zombified cricket. The experimental conditions are as follows:

**target of zombification** Adult crickets (*Gryllus bimaculatu*) that had molted within two weeks before experiments were randomly selected.
**liquid medicine for zombification** mixtures of GABA 1.25 mol/l, Alanine 1.0 mol/l, and Taurine 0.5 mol/l with liquid Ringer’s solution as a solvent
**criteria for zombification** The injected target shows the following behavior within a few minutes after injection; (1) no spontaneous walking, (2) one leg moves periodically when the target is pulled. If zombification fails, the crickets do not move or show spontaneous movement.

Here, our method of zombification (how much liquid medicine should be injected or how much it should be injected) and the criteria for zombification (how to judge whether the cricket becomes a zombie state or not) are ongoing research. Therefore, as a zombification experiment, this verification experiment is a preliminary experiment.

The procedure of the experiment is as follows.

1. Anesthetize a cricket with CO_2_.
2. Set the cricket.
3. Inject 10 ul of the liquid medicine for zombification to the cricket and measure the time taken for injection.
4. Grab the antennae of the cricket with a clip and pull the cricket on a small treadmill with cellulosic paper(Fig. 12). The velocity of the treadmill was 340 mm/min.

**Fig. 12:**
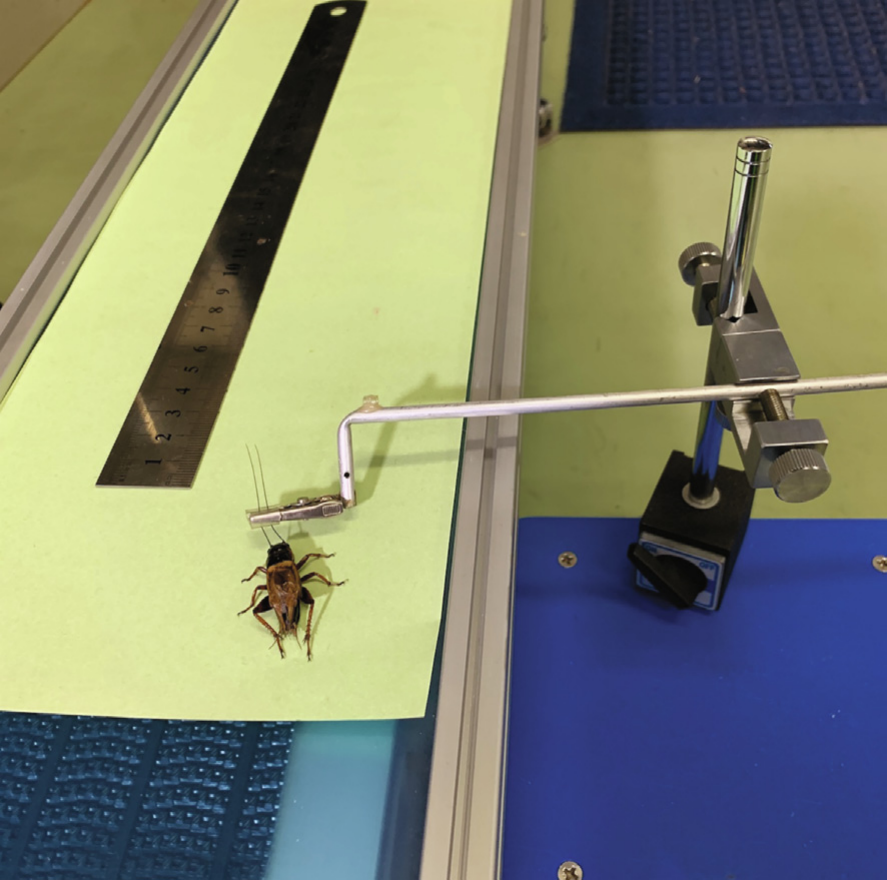
Experimental setup for the verification of zombification. After injection, the antenna of the cricket was held with a small clip and the cricket was pulled by driving the treadmill. Based on the behavior of the cricket during towing, it is judged whether zombification was successful or not.
5. Judge whether zombification was successful according to the above criteria.

The injection experiment by the proposed system was conducted on 20 crickets. The success rate of zombification was 75%, and the average and standard deviation of required time per animal were 93.306±7.302 s. For comparison, the injection by hand was repeated on 20 crickets. In this case, the success rate was 35%, and the average and standard deviation of required time were 50.289±7.569 s. Although the time taken for injection by hand is shorter than that by the proposed system, the success rate when using this system is higher than when doing it manually. These results indicate that the injection time per animal using the proposed system is longer than that of manual injection, but the total time for preparing a large number of injected animals can be shortened due to the high success rate of the proposed system. Therefore, it can be said that this system is sufficiently effective. As described before, the above result is a preliminary experimental result. The success rate of zombification can be improved by advancing our research on zombification methods in the future.

## 8 Conclusion

The proposed microinjection system was designed to support and semiautomate the injections of small biological subjects such as insects in a quick, safe, and consistent manner. The proposed system is not limited to merely injecting crickets or other small animals. Currently, the proposed system only uses OpenCV for capturing still images from the video camera and displaying captured images. By using many software libraries included in OpenCV, multiple-image processing could be implemented, thereby achieving automatic operation by combining image processing with manipulator and syringe-pump operation. In addition, not only can the system be used to inject solutions into biological subjects, it can also be used to conduct electrophysiology experiments to measure the myogenic potential or neuron activity of biological subjects by attaching electrodes to manipulators. Therefore, the proposed microinjection support system shows sufficient extensibility in that it can be applied to other micromanipulations in addition to injections.

In the verification experiment, image processing was not explicitly used, although the proposed system has the capability of realizing an automatic operation based on image processing. The reason is that the targets of injection in the validation experiments were clearly visible. The operation based on the image processing is more effective when the injection target is difficult to see or invisible. On the other hand, it is expected that a broader range of operations can be realized by extending the software of the system, such as image processing. This is an issue for the future.

## Declaration of interest

The authors declare no competing financial interests.

## Human and animal rights

All relevant guidelines were followed.
